# Improvement in the Stability and Enzymatic Activity of *Pleurotus sapidus* Lipoxygenase Dissolved in Natural Deep Eutectic Solvents (NADESs)

**DOI:** 10.3390/life14020271

**Published:** 2024-02-18

**Authors:** Maria Garbe, Leander Tom Lehmann, Ralf Günter Berger, Franziska Ersoy

**Affiliations:** Institute of Food Chemistry, Leibniz University Hannover, 30167 Hannover, Germany; leander.lehmann@stud.uni-hannover.de (L.T.L.); franziska.ersoy@lci.uni-hannover.de (F.E.)

**Keywords:** natural deep eutectic solvent (NADES), lipoxygenase, thermostability, piperonal, basidiomycota, *Pleurotus sapidus*

## Abstract

Natural deep eutectic solvents (NADESs) can serve as solvents for enzymes, are biodegradable, and have low toxicities. Eight NADESs with different hydrogen bond acceptors and donors were tested to improve the stability and activity of a lipoxygenase from Basidiomycete *Pleurotus sapidus* (LOX_PSA_). Betaine:sorbitol:water (1:1:3, BSorbW) and betaine:ethylene glycol (1:3, BEtGly) had the best impact on the peroxidation of linoleic acid and the side reaction of piperine to the vanilla-like scented compound piperonal. The yield of piperonal in NADESs increased by 43% in BSorbW and 40% in BEtGly compared to the control. The addition of BSorbW also enhanced the enzyme’s stability at various temperatures and increased its activity during incubation at 60 °C. The demonstrated improvement in lipoxygenase activity and stability indicates versatile applications in industry, expanding the potential uses of the enzyme.

## 1. Introduction

Lipoxygenases (EC: 1.13.11.12; LOXs) are non-heme, mostly iron-containing fatty acid dioxygenases and are ubiquitously present in plants and animals. LOXs catalyze the dioxygenation of polyunsaturated fatty acids (PUFAs) containing a (1*Z*,4*Z*)-pentadiene system, such as linoleic acid (LA) or α-linolenic acid (α-LeA) [1,2]. The simultaneous co-oxidation of various unsaturated compounds was discovered through the observed bleaching of pigments like carotene [3,4] and has recently been described for the transformation of piperine to piperonal and 3,4-methylenedioxycinnamaldehyde [5].

**Scheme 1:**
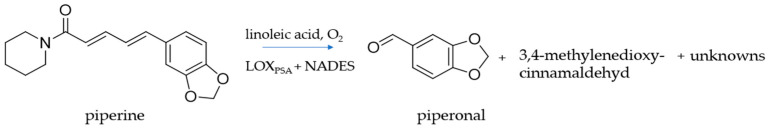
Co-oxidation of piperine to piperonal with LOX_PSA_.

Piperonal (3,4-methylenedioxybenzaldehyde or heliotropin) is an aromatic aldehyde of high interest in the fragrance and flavor industry. It has a sweet, flowery, vanilla-like odor and is present in small amounts in vanilla pods [6,7]. Commercial piperonal was first obtained through alkaline pyrolysis of piperine and oxidation via potassium permanganate, but its price was prohibitively high. A synthesis using safrole as a substrate was equally unsatisfactory because of the associated environmental problems [8]. Biotransformation approaches based on the substrates isosafrole, piperonyl alcohol, or piperonylic acid were established with various enzymes from bacteria and fungal strains [8,9,10]. They were recently extended in a study about a lipoxygenase from *Pleurotus sapidus* that converted piperine, an alkaloid abundant in white and black pepper, to piperonal via a co-oxidative mechanism [5] (Scheme 1). Piperonal received the generally recognized as safe (GRAS) status from the Flavor and Extract Manufactures Association (FEMA) in 1965 and is approved by the FDA for its use in food products [11]. To increase the product yield of piperonal, using so-called natural deep eutectic solvents (NADESs) as the reaction medium was proposed. Such an example can be found in cannabinoid biotransformation.

By adding an NADES, cannabinoid production of tetrahydrocannabinolic acid doubled, and the production of cannabichromenic acid more than tripled [12]. Another example is the production of biodiesel in NADES. Under ideal circumstances, significant conversions of Miglyol^®^ oil were attained with Novozym^®^ 435 in a mixture of choline acetate and glycerol at a ratio of 1:1.5 [13]. NADESs are environmentally friendly and exhibit low flammability and toxicity; they are thus highly suitable for the food and flavor industry [14,15,16]. NADESs can contribute to increasing the product yield by stabilizing the enzymes involved, altering the solubility of substances, or stabilizing substrates [12,17,18,19]. Examples include increases in the chemical stability and catalytic activity of redox enzymes in NADESs containing choline chloride [12,20].

In this article, various NADESs with choline chloride and betaine as a hydrogen bond acceptor (HBA) were tested for their influence on LOX_PSA_ stability at different temperatures, as well as for their impact on the biotransformation of piperine to piperonal and 3,4-methylenedioxycinnamaldehyde to pave the way for an industrial application.

## 2. Materials and Methods

### 2.1. Materials

Chemicals were obtained from Carl-Roth (Karlsruhe, Germany), Sigma-Aldrich (Seelze, Germany) and Merck (Darmstadt, Germany) in *p.a.* quality.

### 2.2. Preparation of NADESs

**Figure 1:**
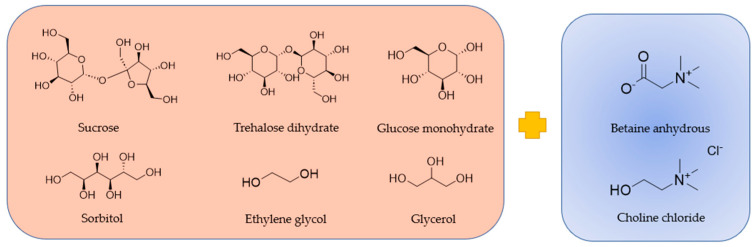
Chemical structures of the NADES components and their compositions.

All NADESs contained betaine or choline chloride as a hydrogen bond acceptor and different combinations of hydrogen bond donors (Table 1 and Figure 1). The components were heated at 70 °C and 300 rpm until the solvents became stable and clear.

### 2.3. Protein Expression and Purification

The *lox1* gene was expressed in *Escherichia coli* DE3 Star cells (Invitrogen, Karlsruhe, Germany) containing the chaperone-encoding plasmid pTf16 as described previously. The recombinantly His-tagged enzyme was purified from the culture supernatant using Ni-NTA affinity chromatography and its purity determined using SDS-PAGE analysis (12% resolving gel, 4% stacking gel) (Figure S1) [5,21,22].

### 2.4. Enzyme Activity Determination

The assay is based on the conversion of linoleic acid into conjugated hydroperoxy fatty acids catalyzed by lipoxygenase. These compounds absorb light at a wavelength of 234 nm, providing a measure of enzyme activity.

To perform the assay, 195 µL of 20 mM sodium phosphate buffer (pH 7) and 5 µL of purified enzyme solution were pipetted into individual wells of a 96-well plate. The reaction was initiated by adding 5 µL linoleic acid (2.5 mM). The incubation took place with regular shaking at 30 °C for 15 min. Using an EON^TM^ microplate reader (BioTek, Winooski, VT, USA), the absorbance was measured, which was then used to calculate the enzyme activity [23]. Triplicate measurements were performed.

### 2.5. LOX_PSA_ Thermostability in Aqueous NADES Media

The thermostability of LOX_PSA_ was tested in sodium phosphate buffer (50 mM, pH 7) with 22% NADES. For the control, 22% Britton–Robinson buffer with a pH value equal to the tested NADES was added. The LOX_PSA_ was incubated at room temperature for 5 min at 200 rpm and at 60, 65 or 70 °C. For the stability tests, samples were taken every 2 min for 10 min. The samples were immediately placed on ice. All samples were brought to room temperature and their activity determined using the assay described above.

### 2.6. Biotransformation of Piperine to Piperonal with LOX_PSA_ in NADES

#### 2.6.1. UHPLC-DAD Measurements

For the biotransformation, 22% NADES (BSorbW or BEtGly), 0.9 mM piperine, 590 µL LOX_SA_ (32 U/mL), 1.5 mM linoleic acid and 100 µL of 20 mM sodium phosphate buffer (pH 7) were added to a 4 mL glass vial. The total volume of the reaction was 1 mL.

As a control, experimental setups with inactive enzyme and with Britton–Robinson buffer (pH adjusted to the pH of the respective NADES) instead of an NADES were used. The mixture was shaken at 200 rpm and 22 °C for 24 h and the reaction was stopped with 1 mL of 99% methanol (HPLC grade) and filtered. As an internal standard, 1 µL of 160 mM ferulic acid ethyl ester was added. The measurements were performed on an Infinity II UHPLC (Agilent Technologies, Santa Clara, CA, USA) using water + 0.1% formic acid as solvent A and acetonitrile as solvent B. An amount of 1 µL of sample was injected into a Poroshell 120 EC-C18 (2.7 µm, 2.1 × 100 mm). The gradient used was 95% A, 0–2 min; 95 to 85%, 2–7 min; 85 to 50%, 7–13 min; 50 to 20%, 13–14 min; 20%, 14–16 min; 20 to 95%, 16–18 min; and 95% A, 18–20 min. The flow rate was set to 0.5 mL/min and the column oven temperature to 25 °C. The chromatograms were evaluated at 354 nm using a DAD detector.

#### 2.6.2. GC-TDS-MS Measurements

Detection and quantification of piperonal was performed via GC analysis after extraction using Twisters (10 × 0.5 mm, Gerstel, Mülheim, Germany) coated with polydimethylsiloxane [5]. For the reaction, 22% NADES (BSorbW or BEtGly), 17 µM piperine, 890 µL LOX_PSA_ (32 U/mL), 27 µM linoleic acid and 100 µL of 20 mM sodium phosphate buffer (pH 7) were added to a 4 mL glass vial. The total volume of the reaction was 2 mL. The mixture was shaken at 200 rpm and 22 °C for 24 h, and the was reaction stopped with 100 µL of 37% HCL and neutralized with NaOH. The sample was mixed with 4 mL of deionized water containing 4.1 μg/mL 1,2-dimethoxybenzene as an internal standard (IS), and extracted for 1 h under vigorous stirring at 300 rpm. Afterwards, the Twister was rinsed with deionized water, dried with a lint free cloth, and analyzed via gas chromatography. The stir bar was measured via TDS3 (thermodesorption system)-GC-MS (Agilent 6890N, Agilent Technologies, Santa Clara, CA, USA). Piperonal was semi-quantified according to the area of the IS. GC mass spectrometry was used for identification (Agilent 6890 N, equipped with a DB-5MS UI column (30 m × 0.25 mm, 0.25 μm, Agilent)), along with CIS 4 coupled with mass spectrometry (mass range 50.00–550.00, scan speed 781 u/s, frequency 2.7 scans/s). GC–MS analyses were performed with helium as a carrier gas, a 0.25 μL injection volume and a flow rate of 1.3 mL/min, and a cold injection system was used (CIS 4, Gerstel). MS scans were run in the range of *m*/*z* 34–500 with a scan rate of 3.1 scans/s. The GC system had an initial temperature of 40 °C (hold time: 3 min) and this was increased up to 300 °C at a rate of 10 °C/min for 34 min (hold time: 5 min) [5].

## 3. Results

### 3.1. LOX_PSA_ Activity Improvement Using NADESs

**Figure 2:**
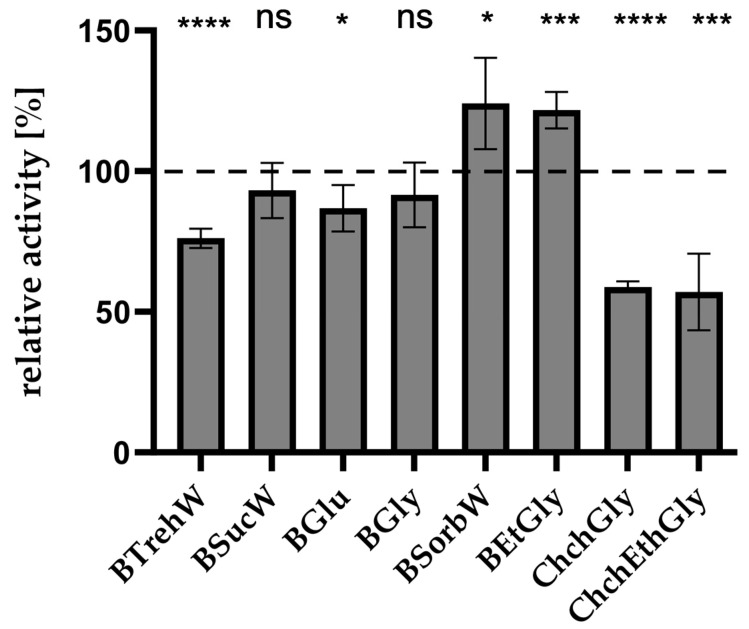
The relative activity of LOX_PSA_ in different NADESs. The activity of LOX without an NADES was determined at the optimum pH (pH 7) for reference (100%). n = 4. Statistical evaluation was carried out using unpaired *t*-tests in comparison to the control, * for *p* < 0.05, *** for *p* < 0.001 and **** for *p* < 0.0001, ns—not significant.

The influence of various natural deep eutectic solvents (NADESs) on the enzyme activity of LOX_PSA_ was investigated (Figure 2). The use of choline chloride as a hydrogen bond donor (HBD) resulted in a loss of activity of about 40%, independent of the tested hydrogen bond acceptor. All tested NADESs with betaine showed only a minor activity loss or increased activities. Sorbitol or glycerol and ethylene glycol in combination with betaine led to significantly higher activities of LOX_PSA_ with 131 (BSorbW) and 122% (BEtGly) respectively. Both significantly surpassed the control value despite the deviation from the optimum pH of 7 (pH of BSorbW: 10.1, BEtGly: 9.7). The addition of sugars such as glucose or sucrose did not lead to a significant increase in enzyme activity. The lowest activity for betaine-based NADESs was observed with BTrehW (76%). For all further experiments, BSorbW and BEtGly were employed as the most promising NADES option.

### 3.2. Enzyme Stability in NADES

**Figure 3:**
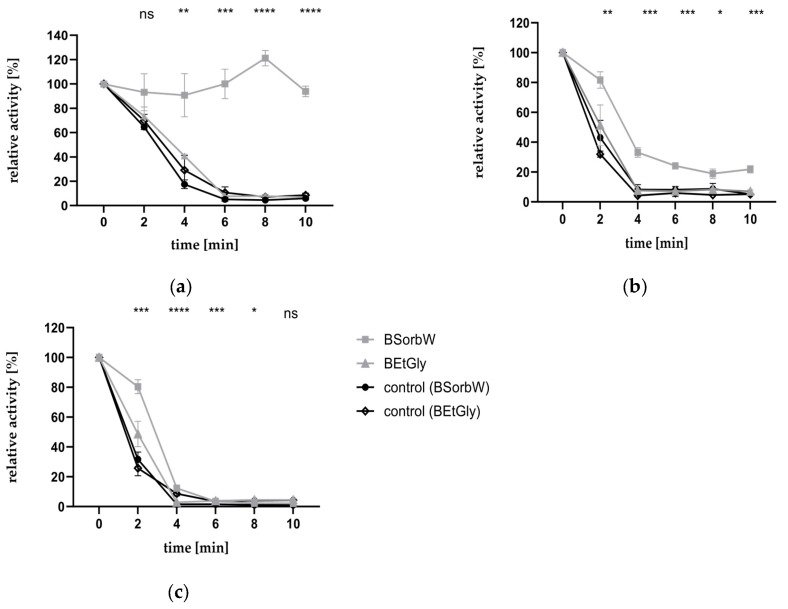
Activity of LOX at various temperatures in 22% NADES (BSorbW or BEtGly), with control activities measured in Britton–Robinson buffer at the same pH as the NADES (BSorbW: pH 10.1, BEtGly: pH 9.7). (**a**) 60 °C, (**b**) 65 °C and (**c**) 70 °C, n = 3. Statistical evaluation was carried out using unpaired *t*-tests in comparison to the control for BSorbW samples. For BEtGly, no significance could be determined. It was therefore not included in the figure. * for *p* < 0.05, ** for *p* < 0.01, *** for *p* < 0.001 and **** for *p* < 0.0001, ns—not significant.

The results showed that the use of BSorbW led to an improvement in the stability of the LOX_PSA_ enzyme at different temperatures compared to the control (Figure 3). At 60 °C, there was a slight (10%) reduction in activity within the first 4 min; after 8 min, the activity increased to 121% (Figure 3a). This indicated a stabilizing effect of BSorbW on LOX_PSA_. For both controls, as well as the sample in BEtGly, there was a decrease in activity to 5 to 10% within 6 min at 60 °C. In BEtGly, the LOX activity was only slightly increased (41 instead of 25% in the control) within the first 4 min at 60 °C. Any stabilizing effect of BEtGly was thus minimal at best at all tested temperatures.

Even at 65 °C, BSorbW achieved enzyme stabilization. There was a decrease in activity within the first 4 min to 33% (Figure 3b). Subsequently, the relative activity remained constant between 19 and 24%. In the control, a significant loss in activity was observed, with a relative activity of only 4% after 4 min. No stabilizing effect was observed in BEtGly at 65 °C.

At 70 °C, BSorbW showed only a slightly stabilizing effect compared to the control and BEtGly (Figure 3c). Within the first 2 min, there was a decrease in activity to 80% in BSorbW, while the values were significantly lower, at 49 (control buffer BSorbW), 32 (BEtGly), and 26% (control buffer BEtGly) in all other samples. After 4 min at 70 °C, the activity decreased significantly in all samples, leading to a complete loss of activity.

Overall, only BSorbW has a significantly stabilizing effect on LOX_PSA_, which was more pronounced at lower temperatures.

### 3.3. Biotransformation of Piperine to Piperonal with LOX in NADESs

**Figure 4:**
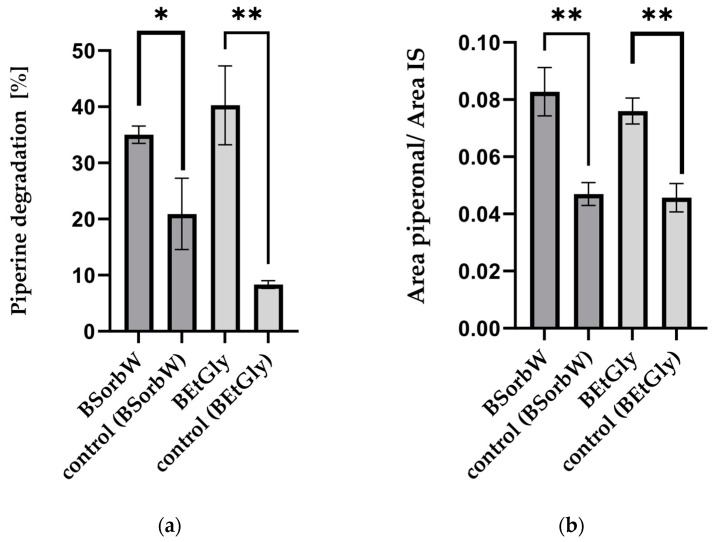
Biotranformation of piperine to piperonal using LOX_PSA_ (co-oxidative reaction with linoleic acid), (**a**) piperine degradation according to UHPLC-DAD measurements at 354 nm and (**b**) semi-quantified piperonal yield relative to the internal standard according to TDS-GC-MS (TDS) measurements. n = 3. Statistical evaluation was carried out using unpaired *t*-tests in comparison to the respective control, * for *p* < 0.05, ** for *p* < 0.01.

The results from the HPLC measurementsdemonstrated an enhanced degradation of piperine within 24 h in both NADESs (Figure 4a). LOX_PSA_ in BSorbW degraded 35% of piperine, while it was able to degrade 40% in BEtGly. In the control groups with the same pH, a significantly reduced degradation of 21 and 8.3%, respectively, was observed. This corresponded to an increase in degradation by a factor of 1.7 in BSorbW and 4.8 in BEtGly.

Regarding the formation of piperonal, the same positive effect upon addition of an NADES was observed, but the difference between the two NADESs was negligible (Figure 4b). The piperonal yield was increased by 43% in BSorbW and by 40% in BEtGly compared to the respective controls. This corresponded to a production of 1.52 µg/mL (10.1 µM) piperonal in BEtGly and 1.66 µg/mL (11.1 µM) in BSorbW (semi-quantified according to the internal standard).

## 4. Discussion

The results demonstrate a positive influence of NADESs on the stability of LOX_PSA_ at various temperatures, an improved activity, and an increased yield of piperonal during the co-oxidation of piperine with linoleic acid. Particularly, using betaine as an HBA led to improved activities and stabilities of LOX_PSA_. In contrast, using choline chloride as an HBA reduced the relative activity of LOX_PSA_ by about 40%, independent of the hydrogen bond donor (Figure 2). So far, choline-chloride-based NADESs have only marginally proven suitable for protein stabilization [24,25]; they are more commonly used in extractions, of, e.g., phenols or metabolites [26,27,28]. It is known that the hydroxylated alkyl chain of choline increases its propensity to form H bonds [29,30]. The choline ion binds to negatively charged aspartic and glutamic acid residues, but also makes substantial contacts with arginine, glutamine, glycine, leucine, methionine, or tyrosine in peptides and proteins [19]. Therefore, ionic liquids with ChCl are highly effective in modifying the stability and activity of enzymes [29]. Combined with ethylene glycol or glycerol in an NADES, this positive effect was not observed for LOX_PSA_. One reason might be the altered interaction of ChCl with the HBD. Due to the diminished or consistent activity of LOX in ChCl-based NADESs, betaine was tested as an additional HBA.

The positive effect of betaine as an NADES component on enzyme stability has already been demonstrated by several studies and was confirmed for LOX_PSA_. BSorbW enhanced the activity of LOX_PSA_ at 60 °C and minimized the activity loss at 65 and 70 °C within the first 4 min (Figure 3). Likewise, NADESs consisting of betaine and sorbitol enhanced the thermal stability of *β*-lactoglobulin. In the temperature range of 27 to 127 °C, an improved protein stability was achieved through the formation of hydrogen bonds [31].

However, not only the HBA is crucial for improved enzyme activity. The combination of an HBD and an HBA influences the viscosity of the NADES and thereby the interaction with the protein. The results of this study indicate that the addition of sugars such as glucose, trehalose, and sucrose did not positively affect the activity of LOX_PSA_. NADESs with ChCl, fructose, and water enhanced the activities of porcine or resin lipases, but showed an inhibitory effect on other lipases [32]. The activity and stability effects can be attributed to the strong polarity due to the multiple hydroxy groups of sugar-based NADESs. The presence of two extra hydroxyl groups in ChCl:xylitol led to a higher viscosity compared to ChCl:glycerol at room temperature [33]. Therefore, in an effort to reduce the viscosity of sugar-based NADESs, a sugar HBD with fewer hydroxyl groups such as fructose should be chosen instead of glucose [14].

The addition of sorbitol/water and ethylene glycol with betaine increased the LOX activity (Figure 2). A study by Xie and Timasheff likewise demonstrated the positive effect of sorbitol on ribonuclease A by altering the folding enthalpy through sorbitol addition in conjunction with thermal unfolding [34]. Gajardo-Parra et al. showed the increased enzymatic activity of horseradish peroxidase in a system of betaine:sorbitol:water. This effect could now be confirmed for betaine as an HBA in combination with LOX.

Another important factor is the content of water in the NADES. In the NADES with sorbitol and betaine, water was added. The water content is important for enzymatic mobility in the system. Added water can also promote interactions that could change the enzyme conformation [35]. The hydration shell of a protein has an influence on enzyme conformation during storage and reactions. The combination of betaine, sorbitol, and water with their positive properties for proteins resulted in an increase in the relative activity (Figure 2). Nevertheless, the combination of betaine and ethylene glycol also showed positive results for the relative enzyme activity of LOX_PSA_. So, co-solvents like NADESs or ionic liquids influence the enzymatic stability and activity by modifying the chemical structure, polarity, and viscosity, and the ability to build hydrogen bonds with the protein surface [36,37].

Regarding the stability of LOX_PSA_ under heat stress, only the combination of betaine:sorbitol:water increased its stability. Betaine:ethylene glycol behaved similarly to the control, with activities dropping rapidly at different temperatures. Particularly noteworthy was the increase in enzyme activity at 60 °C observed after 8 min in BSorW, while the controls and LOX_Psa_ in BEtGly showed a quick loss of activity. One reason for this, as described above, could be the addition of water and the formation of hydrogen bonds to stabilize the enzyme in the NADES–enzyme complex. Gajardo-Parra et al. demonstrated that the addition of NADESs like BSorbW led to an increase in the unfolding temperature of horseradish peroxidase (HRP). The results indicated that interactions between NADESs and amino acids promote the intermediate state during HRP folding and are linked with changes in the secondary structure of the protein. This is accompanied by a slow unfolding at high temperatures; the enzyme exhibits a boost in activity. The enzymatic activity increases due to the exposure of the heme pocket. This positive effect diminishes as the temperature increases, which was also the case for LOX [37].

At 65 and 70 °C, there was no noticeable increase in activity. Nevertheless, the activity of LOX_PSA_ decreased significantly more slowly than in the controls. Within the first 2 min at 70 °C, there was a reduction in activity to 80% in BSorbW in contrast to 26% in the control. This fits well to the effects described above for HRP. In studies about *ß*-lactoglobulin in NADESs (betaine/sorbitol), this decreasing positive effect of NADESs with increasing temperatures was also observed. At higher temperatures, an increased number of betaine molecules interact with the protein through electrostatic interactions, which leads to destabilization of the protein [31].

For the co-oxidation of piperine to piperonal using LOX_PSA_, an increase in the product yield of about 40% was achieved in both NADESs (Figure 4). Due to the co-oxidative character of the reaction, which stage of the reaction was improved through the addition of an NADES cannot be precisely determined. The stabilization of the enzyme through hydrogen bonding during the 24 h reaction period could be a reason for the improved degradation of piperonal. Krahe et al. achieved the generation of up to 25 μM piperonal with the same enzyme in an aqueous buffer at the enzyme’s pH optimum of 7 [5]. In the present study, 44 (BSorbW) and 40% (BEtGly) of this piperonal concentration were reached. The reduction is mainly attributable to the reduced amount of linoleic acid that was used in the present study (1.5 instead of 2.5 mM). A similar reduction in the amount of linoleic acid led to a comparable decrease in the piperonal yield of about 50% in the results published by Krahe et al. This research group also found that that 37 °C was considered the optimal temperature for piperonal synthesis with LOX_PSA_ [5]. In the present experiments, a reaction temperature of 22 °C was chosen to keep the NADES stable. The yields in aqueous buffer at optimal pH and temperature and those in the tested NADES were thus similar under similar reaction conditions. The positive effect of the NADES is most likely balanced by the activity decrease due to the lower pH. As discussed before, the use of an NADES with a pH closer to the pH optimum of LOX will most likely increase the piperonal yield.

Similar results for increasing product yields have been achieved in cannabinoid production. Compared to aqueous systems, the use of NADESs resulted in a twofold increase in cannabinoid production with a tetrahydrocannabinolic acid synthase and more than a threefold increase with a cannabichromenic acid synthase [12]. Gorke et al. reported that upon addition of 10% ChCl:glycerol, the hydrolysis of *p*-nitrophenyl acetate was accelerated moderately when catalyzed by several esterases [38]. Also, for lipase-catalyzed transesterification of ethyl valerate with butan-1-ol using eight different DESs, the biocatalytic reactions were improved significantly (up to 20-fold) [38]. The addition of BEtGly did not have an impact on the enzyme’s stability. It thus probably contributed to promoting the substrate reaction, thereby increasing the yield of piperonal. Nian et al. showed that the reaction of *Candida Antarctica* lipase B (CALB) was activated in NADESs because H bonding increased the size of the acyl-binding pocket and allowed easier access to the substrates. In addition, H bonding increased the nucleophilic properties of the substrates, thus resulting in an increase in CALB activity [39].

An influence of the water content or the pH of the NADES is also possible. A study conducted by Panic et al. utilized NADESs as solvents for plant cell biocatalysis. They successfully reduced 1-(3,4-dimethylphenyl)ethanone to the corresponding enantio-enriched alcohol using carrot roots. The NADESs utilized in this study were prepared at varying molar ratios of choline chloride and glucose, xylose, xylitol, glycerol, or ethylene glycol. The water content in the NADESs influenced the selectivities; 30% (*v*/*v*) of the eutectic solvent consisting of choline chloride and glucose resulted in the (*R*)-enantio-enriched alcohol. However, increasing it to 80% (*v*/*v*) predominantly led to the observation of the (*S*)-antipode [40]. The multistep reaction involved in the biotransformation of piperine to piperonal could also be influenced by the tested NADESs.

## 5. Conclusions

This work demonstrates the high potential of NADESs for industrial applications. By improving the protein stability and increasing product yields, the utility of enzymes can be expanded. The use of BSorbW led to an enhanced enzyme stability at various temperatures, with an increase in the enzymatic activity at 60 °C. Additionally, the tested NADESs, BSorbW and BEtGly, contributed to increased piperonal yields in the co-oxidation cleavage of piperine. Natural piperonal is an expensive flavoring agent in the food industry; thus, enhanced product yields in enzymatic reactions may significantly lower the cost of the final biotransformation product. Moreover, NADESs, due to their low toxicity, are well suited for the food industry. Betaine and sorbitol can be used in foods or are already in use; e.g., sorbitol is used as a sweetener. Similarly, the product yield of piperonal was increased by 40%. This results in a significant cost reduction on the industrial scale and may favorably compete with the natural extraction of piperonal from plant sources.

## Figures and Tables

**Table 1:** NADES compositions, including the molar ratios and abbreviations.

Abbreviations	Component A	Component B	Component C	Molar Ratio
BTrehW	Betaine anhydrous	Trehalose dihydrate	Water	4:1:14
BSucW	Betaine anhydrous	Sucrose	Water	2:1:10
BGlu	Betaine anhydrous	Glucose monohydrate	-	5:2
BGly	Betaine anhydrous	Glycerol	-	1:2
BSorbW	Betaine anhydrous	Sorbitol	Water	1:1:3
BEtGly	Betaine anhydrous	Ethylene glycol	-	1:3
ChChGly	Choline chloride	Glycerol	-	1:2
ChChEtGly	Choline chloride	Ethylene glycol	-	1:3

## Data Availability

The datasets used and analyzed during the current study are available from the corresponding author upon reasonable request.

## Supplementary Materials

The following supporting information can be downloaded at: https://www.mdpi.com/article/10.3390/life14020271/s1. Figure S1: Purification of the recombinant LOX_PSA_ by Ni-NTA affinity chromatography. SDS-PAGE stained with Coomassie Brilliant Blue.
