# Supplementary material for: Improvement in the Stability and Enzymatic Activity of Pleurotus sapidus Lipoxygenase Dissolved in Natural Deep Eutectic Solvents (NADESs)
Source: Life (Basel). 2024 Feb 18;14(2):271. doi: 10.3390/life14020271 (PMC10890681; doi:10.3390/life14020271)

Supplementary data

Figure S1: Purification of the recombinant $LOX_{PSA}$ by Ni-NTA affinity chromatography. SDS-PAGE stained with Coomassie Brilliant Blue.1 and 4: non purified culture supernatant 2: $LOX_{PSA}$ before buffering, 3: $LOX_{PSA}$, 5 and 6: washing steps, M: molecular mass marker.

M 1 2 3 4 5 6 M

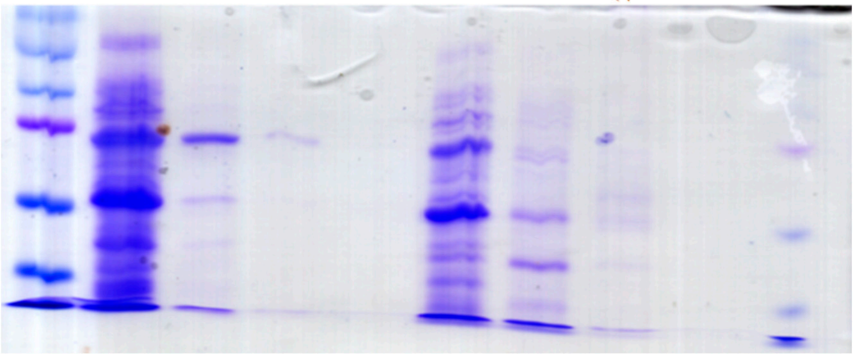

Supplement: Supplementary file 1 [file life-14-00271-s001.zip › life-2869129-supplementary.pdf]
